# Pernicious Anemia and Vitiligo in an HIV Patient: An Unfamiliar Case Presentation

**DOI:** 10.1155/2020/7942453

**Published:** 2020-05-29

**Authors:** Katongo Mutengo, Francis Mupeta, Owen Ngalamika

**Affiliations:** ^1^Department of Internal Medicine, Livingstone Central Hospital, Ministry of Health, Livingstone, Zambia; ^2^Infectious Diseases Unit, Department of Internal Medicine, University Teaching Hospital, Lusaka, Zambia; ^3^Dermatology and Venereology Section, University Teaching Hospital, University of Zambia, School of Medicine, Lusaka, Zambia

## Abstract

Pernicious anemia (PA) is a rarely considered cause of anemia in HIV-infected population and is seldom on the list of differential diagnoses. However, PA can have serious consequences if misdiagnosed or left untreated. We present the case of a 38-year-old HIV-positive man who was diagnosed with PA, which was preceded by a one-year history of vitiligo. Our case is a reminder for clinicians to have a high index of suspicion for an autoimmune process as a potential cause of anemia in HIV-infected individuals.

## 1. Introduction

Anemia is one of the commonest presentations among human immunodeficiency virus- (HIV-) infected individuals. However, due to the diversity of etiological factors in the HIV-infected population such as poor diet, medications, secondary infections, or the virus itself, anemia is rarely attributed to an autoimmune process [1]. More so, in resource-limited settings, the high cost of workup coupled with a low index of suspicion makes it almost impossible to investigate for autoimmune causes of anemia such as PA. Yet, if left untreated, PA can have seriously debilitating effects including irreversible neurologic damage and susceptibility to gastric tumors associated with the disease [2].

Scientific evidence shows that autoimmune activation can be triggered by environmental and infectious agents [3], including HIV. But, the decline in immunity associated with HIV would often make the autoimmune process occult while the converse holds true as well [4]. Despite PA generally contributing close to 90% of vitamin B12 deficiency in Africa [5], it is rarely investigated for as a cause of vitamin B12 deficiency in the HIV population, which may present as a macrocytic anemia. In fact, extensive literature research yields scanty information on PA occurring in HIV population to date. The antipathy of PA and HIV disease processes, therefore, presents a diagnostic enigma to clinicians especially when the autoimmune process is coexisting in the setting of a low immune status. Here, we present the case of an HIV-positive Zambian man of African descent diagnosed with PA, who presented with vitiligo a year before the diagnosis of macrocytic anemia. This case highlights the need of screening for an autoimmune process as a cause of anemia even in the presence of an underlying disease with contrasting pathogenesis such as HIV.

## 2. Case Presentation

A 38-year-old Zambian man of African descent presented to Livingstone Central Hospital, a tertiary institution in the southern province of Zambia, with a history of spontaneous loss of skin pigmentation on the face for 20 months and progressive lethargy for 8 months. The skin hypopigmentation started as small pink patches on his cheeks and nose but progressively increased in size. He was said to be in good health, was energetic, and was able to carry out his usual activities despite the facial hypopigmentation. However, about 12 months after the onset of this condition, he described feeling weak and tired on minimal exertion such as walking a distance of about 6 meters. He, however, denied any history of constitutional symptoms, cough, body swelling, orthopnea, paroxysmal nocturnal dyspnea, or joint pains. He also denied any history of sensory paresthesias. He was diagnosed with HIV-1 about 4 years prior to presentation. However, he initially declined initiation of combined antiretroviral therapy (cART) and opted for traditional herbal medication which he took for 4 years. There was no history of any other comorbidities.

The symptoms of lethargy were ongoing for one month when he first presented to the general practitioner (GP). After a full clinical assessment, a complete blood count was done, and he was found to have a hemoglobin concentration of 6.9 g/dL with a mean cell volume (MCV) of 121 fl and mean cell hemoglobin (MCH) of 42.5 pg/cell. Other parameters on the hemogram were unremarkable. His renal biochemistry and liver biochemistry were normal. He was prescribed folic acid 5 mg once a day by his GP. He was also recounselled and initiated on cART comprising Tenofovir disoproxil fumarate 300 mg once daily, lamivudine 150 mg once daily, and dolutegravir 50 mg once daily (TLD). His baseline CD4 count at the start of cART was 350 cells/*μ*L, and HIV viral load was 76,118 copies/ml. No further workup was done. He continued on this treatment for 7 months. However, despite this therapy, he stated that he continued getting weaker, and the weakness was throughout the day until he was no longer able to get up from his sitting position and often required help to move. He was, therefore, referred to our medical clinic for further assessment.

**Figure 1:**
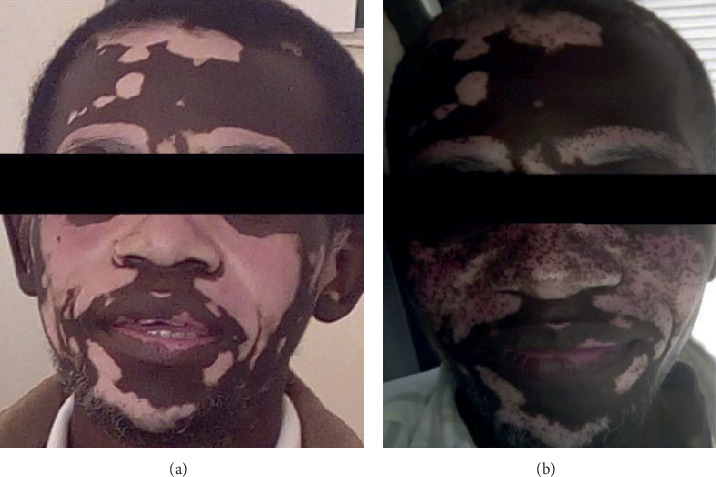
(a) Before treatment with parenteral vitamin B12. Hypopigmented, well-demarcated patches on the nose, lips, cheeks, eyebrows, part of the ears, and forehead in a symmetrical distribution. (b) Repigmentation of affected parts of the skin 3 weeks after treatment.

On examination, he was of fair nutritional status and was lethargic and breathless at rest. He could not get up from his sitting position without assistance. His blood pressure was 123/88 mmHg in an erect position, radial pulse was 112 beats per minute, respiratory rate was 24 breaths per minute, and peripheral oxygen saturation was 98% at room air. He had obvious conjunctival and palmar pallor, but there was no icterus, hyperpigmentation of palmar creases, lymphadenopathy, or peripheral edema. The oral cavity was normal. Local examination revealed hypopigmented, well-demarcated patches on the nose, lips, cheeks, eyebrows, part of the ears, and forehead in a symmetrical distribution consistent with vitiligo (Figure 1(a)). Examination of the lower limbs did not reveal any dysesthesia, proprioception disturbance, spastic paraparesis, or tetraparesis. Further systemic examination was only significant for a resting tachycardia on the cardiovascular system with a heart rate of about 110 beats per minute. Subsequent laboratory workup was noteworthy for severe macrocytic anemia with a hemoglobin level of 3.9 g/dl and MCV of 115.5 fl, very low serum vitamin B12 levels at <61 pmol/l, and positive intrinsic factor (IF) antibodies test. His repeat CD4 count was 920 cells/*μ*L, and HIV viral load was undetectable. Endoscopy and biopsy of the gastric mucosa were not done due to nonavailability of the service at our institution at the time of presentation. A summary of laboratory results before and after treatment is shown in Table 1.

A diagnosis of pernicious anemia was made based on the results, and the patient was started on vitamin B12 (hydroxocobalamin) injections at 1000 mcg once daily for one week, followed by 1000 mcg weekly for 4 weeks and thereafter 1000 mcg once monthly for life. He was also started on prednisolone 40 mg once daily for 5 days, tapered down by 10 mg every 5 days, and finally maintained at 5 mg once daily, not exceeding 3 months. He showed marked improvement in his serum vitamin B12 levels after 3 weeks of treatment as well as improvement on his hemogram (Table 1). There was also notable repigmentation of the parts of skin affected by vitiligo (Figure 1(b)). Our patient was able to resume his normal duties 3 weeks after treatment.

## 3. Discussion

PA, also known as Biermer disease or Addison anemia, is an autoimmune disease presenting as vitamin B12 (cobalamin) deficiency [2]. It results from a deficiency of intrinsic factor (IF), a protein responsible for binding and transport of dietary cobalamins [2, 6]. Studies indicate that PA is the commonest cause of vitamin B12 deficiency worldwide, and in Africa, PA contributes close to 90% of vitamin B12 deficiency [5, 7]. It is, however, uncommon to find PA as a cause of vitamin B12 deficiency in HIV as was the case with our patient.

Generally, anemia in HIV is attributed to poor appetite accompanying chronic illness, coinfections such as tuberculosis, and drugs such as zidovudine, co-trimoxazole, and fluconazole commonly used in the management of HIV [1]. More so, in resource-limited settings, the diagnosis of PA is likely to be overshadowed by a low index of suspicion as well as the lack of readily available resources for investigations. With the coexistence of immunosuppressive conditions such as HIV, the suspicion index may further be masked by the fact that the two conditions require two disparate pathways for their pathogenesis.

Infections have been known to trigger immune activation leading to autoimmune diseases [3]. Therefore, HIV, as an infectious etiology can also trigger an autoimmune process. However, the decline in immunity associated with HIV would often make the autoimmune process occult [4]. It would be expected that unmasking of the autoimmune disease process would often occur after the resurgence of immunosuppressive status. In the case of PA, autoimmunity has been postulated to be initiated by pathogenic autoreactive CD4 T-lymphocytes which do not express CD25 receptors, a T-cell-activation marker expressed by regulatory T-lymphocytes. These gastric-derived pathogenic autoreactive CD4 T-lymphocytes are specifically directed against H+/K+ ATPase pump and IF [6, 8]. Large populations of these pathogenic autoreactive CD4 T-lymphocytes have been found infiltrating the gastric parietal cells in PA leading to autoimmune gastritis by promoting local cytotoxicity and B-cell immune activation [8]. This case may, therefore, shade light on immune dysregulation associated with HIV, especially that CD4 T-lymphocytes are the major target for HIV-1 infection [9]. Studies have shown that gut-associated lymphoid tissue (GALT) is a site of early viral seeding and establishment of the proviral reservoir which causes a distorted activation pattern of lymphocytes [10]. Our patient had a CD4 count of 350 cells/*μ*L with an HIV viral load of 76,118 copies/ml when he first presented. This would place the patient in stage II HIV disease (CD4 count of 200–499 cell/*μ*L), in which according to Zandmann-Goddard et al., autoimmune diseases are unlikely [4]. Could it be possible, therefore, that the GALT HIV-1 infected CD4 T-lymphocytes may have been the pathogenic autoreactive CD4 T-lymphocytes in this patient? Indeed, more studies are needed.

The anemia in our patient was highly attributed to PA due to positive intrinsic factor antibodies (IFA), low levels of vitamin B12, macrocytosis, and co-occurrence of vitiligo. This was further consolidated by the improvement of clinical and laboratory parameters with parenteral vitamin B12 replacement. Scientific evidence also indicates the frequent association of PA with other autoimmune diseases such as autoimmune vitiligo, thyroid disease, and type 1 diabetes [2, 11]. This could explain the presence of vitiligo in our patient. Our patient was further screened for thyroid hormone disorders, diabetes mellitus, and Addison's disease, but the tests were normal.

In the gastric mucosa of PA patients, there is a high proportion of autoreactive activated CD4+ T cells that specifically recognize intrinsic factor [2, 8]. Hence, there is a strong association between PA and autoimmune gastritis which may progress to gastric atrophy and may eventually result in malignant transformation [8, 12]. It is therefore highly recommended to perform an endoscopy and biopsy to diagnose or rule out these changes. On the other hand, *Helicobacter pylori* (*H. pylori*) infection is associated with both PA and vitamin B12 deficiency [2, 8, 13]. This association is reported to result from the triggering of autoantibodies by a mechanism of molecular mimicry. A direct test on an endoscopic biopsy would have led to the definitive diagnosis of *H. pylori* in our patient. Unfortunately, endoscopy was not available at our center at the time the patient was seen. Nevertheless, there was enough evidence to suggest a diagnosis of PA in our patient.

Our patient responded well to parenteral vitamin B12 and steroid therapy. Vitamin B12, given as hydroxocobalamin, has also been shown to reduce the gastric inflammatory process as well as retard the development of immune dysfunction associated with HIV/AIDS [14, 15]. On the other hand, short course steroid therapy was given as adjunct treatment, as it has been shown that corticosteroids improve vitamin B12 absorption by reducing gastric inflammation [16]. Steroid therapy, given as a short term course of not more than 158 days, has been known to be well tolerated and reasonably safe in HIV infection [17].

At his latest review 3 weeks later, our patient had shown great improvement in both his clinical and laboratory markers. He reported complete resolution of the clinical symptoms of anemia as well as partial repigmentation of the skin and had since gone back to his usual activities. He was counselled on the need for lifelong vitamin B12 injections and need for twice-yearly endoscopy for gastric malignancy surveillance at a different institution providing the service in a different town.

Our case is a reminder of the likely cause of macrocytic anemia in the HIV population. A perfunctory view in most of our healthcare settings showed that the immediate care plan for a patient presenting with macrocytic anemia is usually folic acid replacement as was the case with this patient. However, due to the pathophysiological basis of the disease, parenteral vitamin B12 replacement and not folate is needed in the management of PA to avert deleterious effect. To the best of our knowledge, this is the first case reported of PA in HIV infection in Zambia. We, therefore, recommend an autoimmune screen in HIV patients who present with either vitiligo alone or in combination with macrocytic anemia.

## 4. Conclusion

PA is a difficult autoimmune condition to diagnose as a cause of anemia especially in the setting of HIV infection. Clinicians need to have a high index of suspicion for PA in patients presenting with macrocytic anemia, as treatment will often require vitamin B12 replacement rather than folate or blood transfusion. Any delay in treatment is detrimental to the patient.

## Figures and Tables

**Table 1:** Laboratory test and results before and after treatment.

	7 months before presentation	At time of presentation	3 weeks after treatment	References
White cell count (×10^9^/L)	4.1	8.0	7.7	4.0–10.0
Hemoglobin (g/dl)	^*∗*^6.9	^*∗*^3.9	^*∗*^11.0	14.3–18.3
Mean cell volume (Fl)	^*∗*^121	^*∗*^155.5	^*∗*^101.4	79.1–98.9
Mean corpuscular hemoglobin (pg/cell)	^*∗*^42.5	^*∗*^35.9	^*∗*^30.5	27.0–32.0
Platelet count (×10^9^/L)	288	^*∗*^107	292	142–375
CD4 count (*μ*L)	^*∗*^350	920		500–1,400
Viral load (copies per ml)	76,118	Undetectable		<20
S-vitamin B12 (pmol/L)	Not done	^*∗*^<61	>1476	>138
Intrinsic factor antibodies (IFA) (AU/ml)	Not done	^*∗*^>153		0.93–<1.20
Parietal cell antibodies (PCA)	Not done	Negative		
TSH (*μ*IU/mL)	Not done	2.14		0.27–4.20
FT4 (pmol/L)	Not done	15.5		10.16–22.0
FT3 (pmol/L)	Not done	5.0		2.8–12.0
Serum 9am cortisol (mmol/L)	Not done	295		101–535
Fasting blood glucose (mmol/L)	Not done	5.1		4.0–5.4

^*∗*^Above or below the reference range
